# The cardiac toxicity CMR Study in patients with lung cancer treated with chemo-radiotherapy: The CART study- a semi quantitative analysis of the myocardial perfusion index

**DOI:** 10.1186/1532-429X-18-S1-P129

**Published:** 2016-01-27

**Authors:** Kenneth Mangion, Colin Berry, John Foster, Stefan Nowicki, Naveed Sattar, Noelle O'Rourke, Martin Glegg, Marimuthu Sankaralingham, James Paul, Claire Lawless, Jon Stobo, Nazia Mohammed, Aleksandra Radjenovic

**Affiliations:** 1 grid.8756.c000000012193314XBHF Glasgow Cardiovascular Research Centre, University of Glasgow, Glasgow, UK; 2 grid.413301.40000000105239342Clinical Physics, NHS Greater Glasgow and Clyde, Glasgow, UK; 3 grid.413301.40000000105239342The Beatson West of Scotland Cancer Centre, NHS Greater Glasgow and Clyde, Glasgow, UK; 4 grid.8756.c000000012193314XCancer Research UK Clinical Trials Unit, Institute of Cancer Sciences, University of Glasgow, Glasgow, UK

## Background

There are limited data on the cardiac effects of radiotherapy and chemo-radiotherapy on the heart in patients with non-small cell lung cancer (NSCLC). CART is a pilot and feasibility study designed to investigate change in myocardial function and identify changes in myocardial tissue properties in patients being treated with radical radiotherapy. Patients will undergo cardiac magnetic resonance (CMR) exam at baseline, during treatment, at 6 weeks and at 6 months after treatment completion. One component of a multi-parametric CMR protocol is contrast-enhanced first-pass perfusion assessment. Here we report our preliminary findings related to temporal changes in myocardial perfusion index (MPI).

## Methods

CMR was performed on a Siemens MAGNETOM Verio (Erlangen, Germany) 3.0 Tesla scanner. First-pass myocardial perfusion was assessed by saturation recovery prepared dynamic contrast enhanced sequence during administration of 0.1 mmol/kg of gadoterate meglumine (Dotarem). Semiquantitative analysis was performed on segmented basal and mid-LV short axis slices to derive normalized upslopes of myocardial signal intensity profiles (myocardial perfusion index, MPI). The change in MPI over time was analysed statistically using a linear mixed effects model.

## Results

13 patients currently have undergone CMR at baseline and during treatment (mean age 66 years, SD: 9; 71% male), 8 patients have undergone CMR at 6 weeks post treatment initiation (5 patients lost to follow-up/ died), and 6 patients have undergone CMR at 6 months from baseline (n= 2 lost to follow-up/ died).

**Figure 1:**
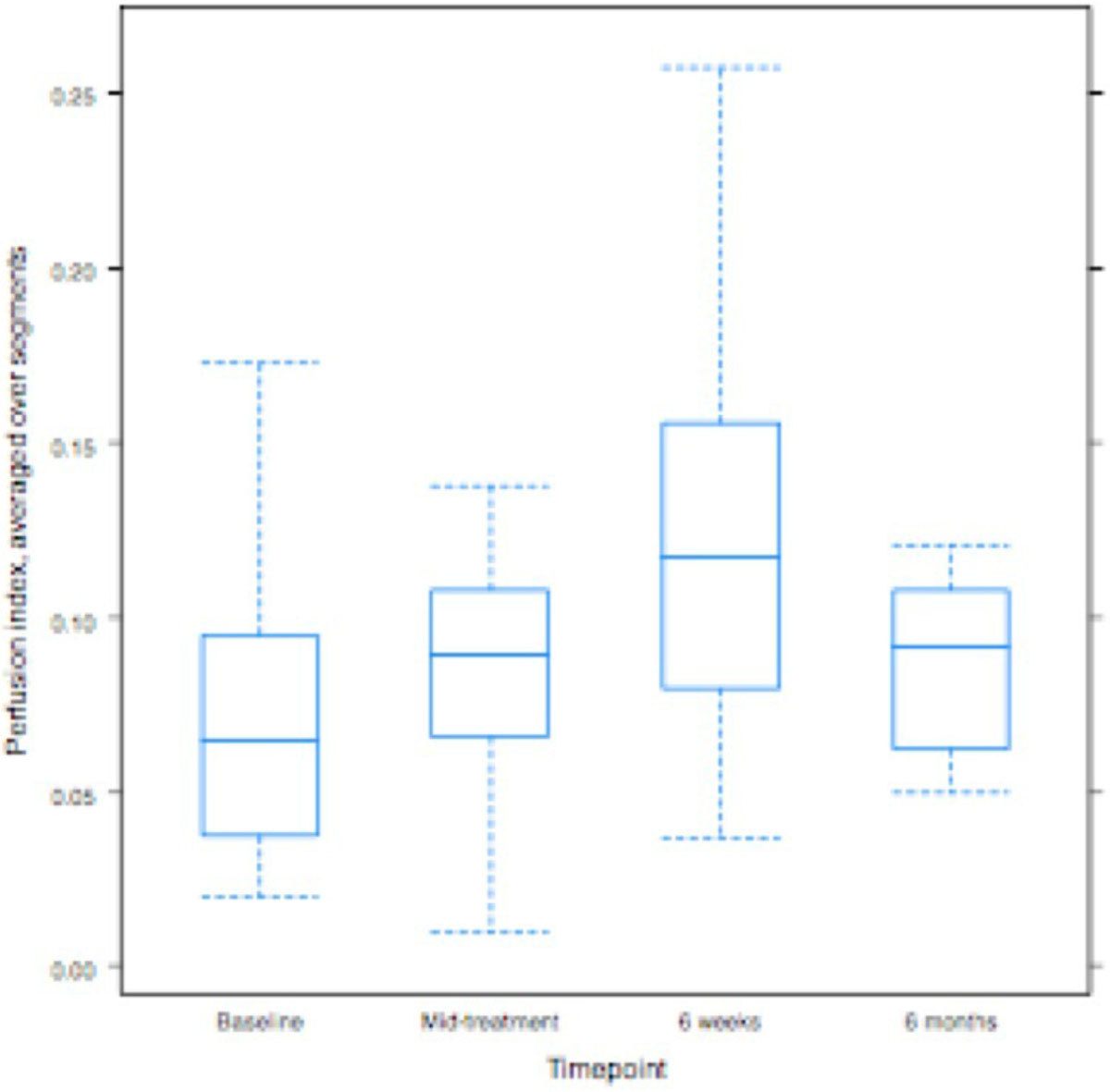
**Boxplots of global myocardial perfusion index values by time point**.

Perfusion index (Figure 1) varies significantly with time (p = 0.0015). After adjustment for multiple testing, the increase from baseline (is statistically significant at 6 weeks (p = 0.014) and approaches significance at 6 months (p = 0.074) post treatment. There is no significant change in perfusion index between baseline and mid treatment (p = 0.75).

## Conclusions

Based on our results, perfusion CMR is a feasible tool to assess changes in microvascular function in patients with NSCLC following radiotherapy/chemo-radiotherapy. We have found that the change in microvascular perfusion is most pronounced 6 weeks following the completion of treatment. This finding will be used in the design of future clinical studies, where measurement of MPI will provide a robust comparison of different existing and emerging treatment protocols for NSCLC with regard to their effects on myocardial physiology.

